# Supplementary figures and images for: Is UA/HDL-C a Reliable Surrogate Marker for Fatty Liver? A Comparative Evaluation with Metabolic Scores in a Mexican Population: The Genetics of Atherosclerotic Disease Study
Source: Diagnostics (Basel). 2025 Jun 3;15(11):1419. doi: 10.3390/diagnostics15111419 (PMC12154243; doi:10.3390/diagnostics15111419)

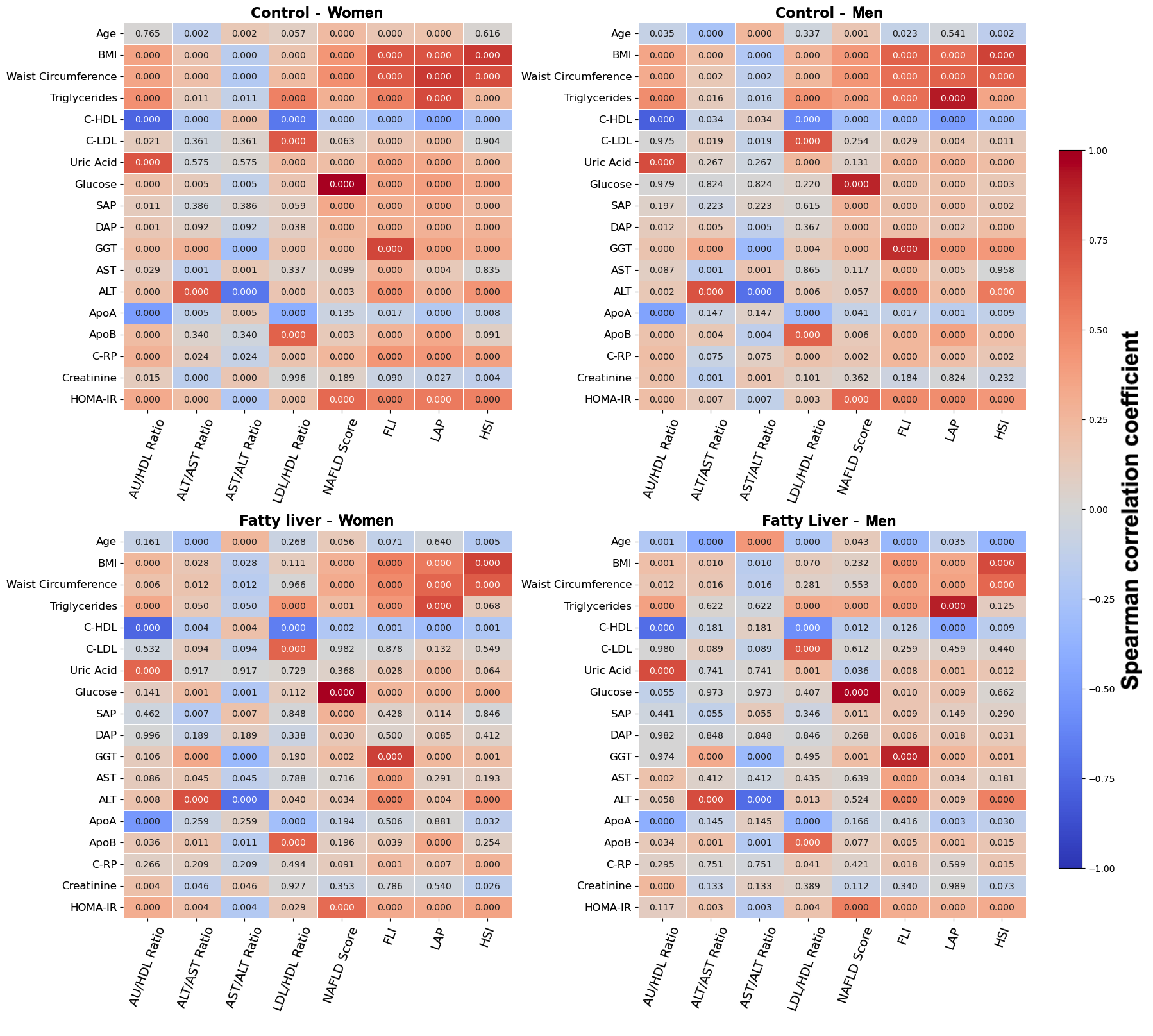

Supplement: Supplementary file 1 [file diagnostics-15-01419-s001.zip › Supplementary data 1.png]

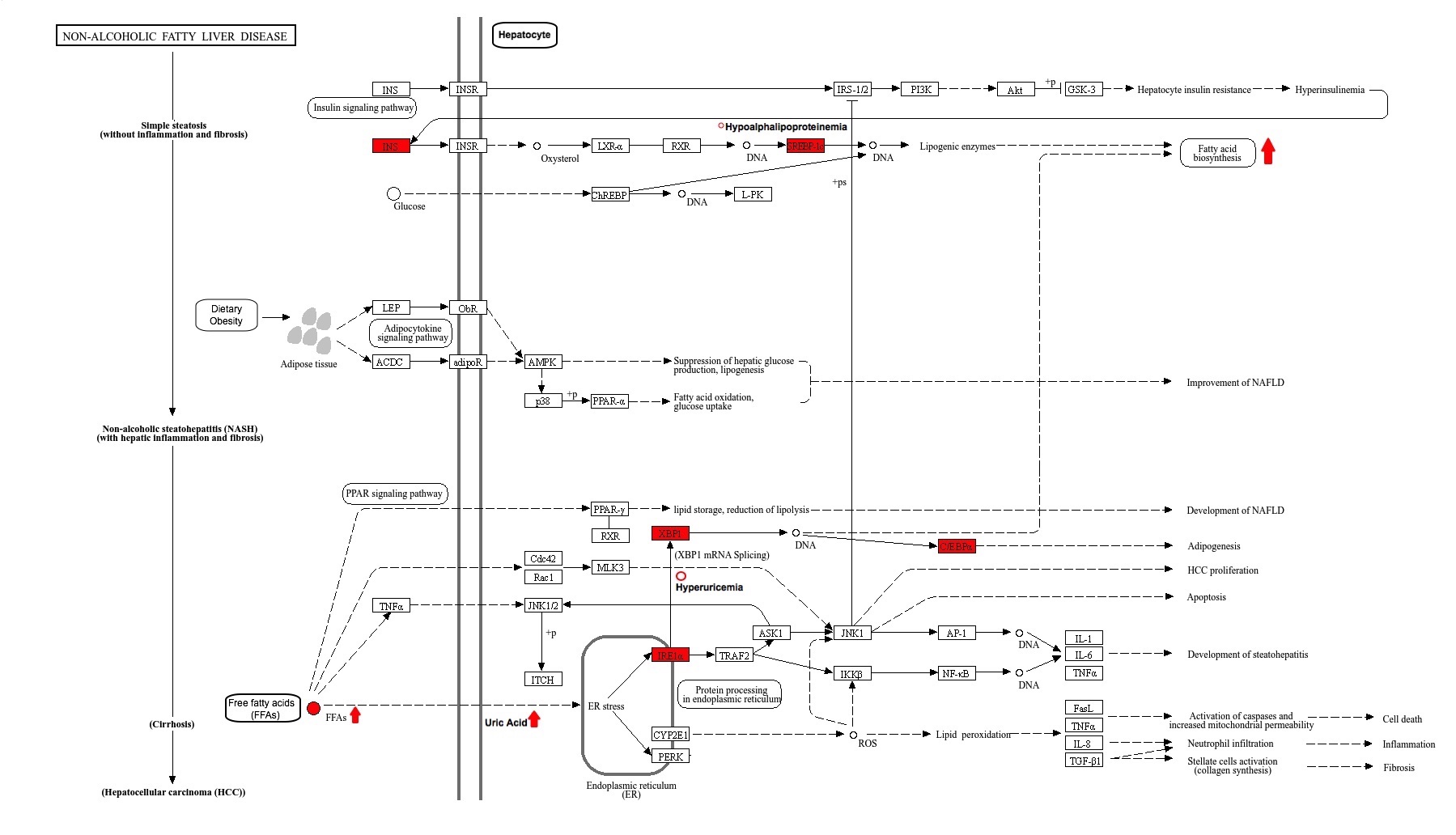

Supplement: Supplementary file 1 [file diagnostics-15-01419-s001.zip › Supplementary data 2.jpg]
